# Molecular features of biguanides required for targeting of mitochondrial respiratory complex I and activation of AMP-kinase

**DOI:** 10.1186/s12915-016-0287-9

**Published:** 2016-08-09

**Authors:** Hannah R. Bridges, Ville A. Sirviö, Ahmed-Noor A. Agip, Judy Hirst

**Affiliations:** Medical Research Council Mitochondrial Biology Unit, Wellcome Trust / MRC Building, Hills Road, Cambridge, CB2 0XY UK

**Keywords:** NADH:ubiquinone oxidoreductase, Respiratory complex I, Metformin, Biguanide, AMP kinase

## Abstract

**Background:**

The biguanides are a family of drugs with diverse clinical applications. Metformin, a widely used anti-hyperglycemic biguanide, suppresses mitochondrial respiration by inhibiting respiratory complex I. Phenformin, a related anti-hyperglycemic biguanide, also inhibits respiration, but proguanil, which is widely used for the prevention of malaria, does not. The molecular structures of phenformin and proguanil are closely related and both inhibit isolated complex I. Proguanil does not inhibit respiration in cells and mitochondria because it is unable to access complex I. The molecular features that determine which biguanides accumulate in mitochondria, enabling them to inhibit complex I in vivo, are not known.

**Results:**

Here, a family of seven biguanides are used to reveal the molecular features that determine why phenformin enters mitochondria and inhibits respiration whereas proguanil does not. All seven biguanides inhibit isolated complex I, but only four of them inhibit respiration in cells and mitochondria. Direct conjugation of a phenyl group and bis-substitution of the biguanide moiety prevent uptake into mitochondria, irrespective of the compound hydrophobicity. This high selectivity suggests that biguanide uptake into mitochondria is protein mediated, and is not by passive diffusion. Only those biguanides that enter mitochondria and inhibit complex I activate AMP kinase, strengthening links between complex I and the downstream effects of biguanide treatments.

**Conclusions:**

Biguanides inhibit mitochondrial complex I, but specific molecular features control the uptake of substituted biguanides into mitochondria, so only some biguanides inhibit mitochondrial respiration in vivo. Biguanides with restricted intracellular access may be used to determine physiologically relevant targets of biguanide action, and for the rational design of substituted biguanides for diverse clinical applications.

**Electronic supplementary material:**

The online version of this article (doi:10.1186/s12915-016-0287-9) contains supplementary material, which is available to authorized users.

## Background

Biguanides are commonly prescribed drugs for the treatment of type II diabetes and to prevent malaria, and are under investigation for their uses in cardiovascular disease and cancer [1–3]. Although their modes of action in all of these applications are still debated, biguanides with known anti-hyperglycemic properties (metformin, phenformin, and buformin) have consistently been observed to inhibit mitochondrial respiratory complex I (NADH:ubiquinone oxidoreductase) at millimolar and high micromolar concentrations [4–6]. Although these inhibitory concentrations appear high, they are physiologically relevant because biguanides are positively charged molecules and are thus concentrated inside mitochondria by the mitochondrial membrane potential. Inhibition of complex I leads to the activation of AMP-activated protein kinase (AMPK), which is thought to contribute to biguanide anti-hyperglycemic activity [7, 8]. Furthermore, biguanides have been found to inhibit the proliferation of cancer cell lines [9, 10] and their mechanisms of action (through mTORC1 in either an AMPK-dependent or independent manner [3, 11]) are suggested to rely on the inhibition of respiratory complex I; overexpression of the yeast alternative NADH:ubiquinone oxidoreductase NDI1 was found to ablate the effect [12]. Conversely, although the antimalarial biguanide cycloguanil and its pro-drug proguanil inhibit isolated complex I, they do not inhibit cellular or mitochondrial respiration [4] and so, unlike the anti-hyperglycemic biguanides, they are not associated with lactic acidosis, a side effect of complex I inhibition. Biguanides thus exhibit a wide range of physiological effects, and it is important to understand the factors that determine their anti-hyperglycemic, anti-proliferative, and anti-malarial effects, and the interplay between these desirable effects and the undesirable side effect of lactic acidosis.

For biguanides to inhibit mitochondrial respiration they must cross both the plasma and mitochondrial inner membrane to reach their binding site on complex I. Metformin transport across the plasma membrane is known to involve organic cation transporters (OCT1–3) and multidrug and toxin extrusion proteins (MATE1–2) [13]. The human thiamine transporter (THTR-2) [14], the serotonin transporter (SERT), and the plasma membrane monoamine transporter (PMAT) have also been implicated in metformin transport [15]. Biguanide uptake is tissue specific due to varying levels of transporter expression [16, 17] and proximity to the hepatic portal vein, owing to the first-pass effect [13]. In contrast to the cellular-level understanding that has been reached about metformin uptake by the intestines and liver, and efflux by the liver and kidneys [13], very little is known about how, once they have entered cells, biguanides enter mitochondria.

There is no consensus yet on whether biguanides that are more hydrophobic than metformin traverse biological membranes by passive diffusion or require protein transporters. Biguanides have high p*K*_a_ values, so they are positively charged at neutral pH, and low partition coefficients, suggesting they are only poorly lipophilic [18]. Even so, many researchers have considered phenformin able to cross biological membranes without requiring active transport [6, 19, 20]. One study concluded that OCT1 mediates the uptake of metformin but not phenformin by rat hepatoma cells [8], because phenformin uptake was not observed to be impaired by the OCT1 inhibitor quinidine. Conversely, the organic cation/carnitine transporter 1 (OCTN1) in the mitochondrial inner membrane [21] was found to contribute to phenformin uptake [22]. Irrespective of the mode of transport, the very different levels of uptake of different biguanides with comparable lipophilicity (such as phenformin and proguanil) [4] argues for their selective transport across the mitochondrial inner membrane.

Here, we aim to determine why some biguanides are able to enter mitochondria and inhibit respiration whereas others (of comparable or greater hydrophobicity) are not, and to determine whether uptake into the mitochondrial matrix is a prerequisite for biguanide-mediated activation of AMPK.

## Methods

### Materials

Phenformin, phenyl biguanide (PubChem CID: 5932) (Sigma-Aldrich Ltd.), isopropyl biguanide (PubChem CID: 9570185), chlorophenformin (PubChem CID: 67587204) (AKOS GmbH), and benzyl biguanide (PubChem CID: 9570091) (Angene International Ltd.) were added from aqueous stock solutions, and proguanil (Sigma-Aldrich Ltd.) and bis-isopropyl biguanide (PubChem CID: 23437065) (European Directorate for the Quality of Medicines and Healthcare) from stock solutions in DMSO.

### Preparation of proteins, membranes, and mitochondria

Complex I and mitochondrial membranes were prepared from *Bos taurus* (bovine) heart [23]. Intact mitochondria were isolated from rat liver [24] and mouse heart and liver [25]. Membranes were prepared from mouse heart mitochondria by 5 s bursts of sonication at 4 °C and collected by centrifugation (75,000 × g, 1 h).

### Kinetic measurements on complex I and mitochondrial membranes

Assays were performed at 32 °C in a SpectraMax 96-well plate reader. NADH:decylubiquinone oxidoreduction by complex I at 0.5 μg mL^−1^ was measured in 20 mM Tris-HCl (pH 7.2), 0.15 % soy bean asolectin (Avanti Polar Lipids), and 0.15 % 3-((3-cholamidopropyl)dimethylammonium)-1-propanesulfonate (CHAPS, Merck Chemicals Ltd), with 200 μM decylubiquinone and 200 μM NADH, and monitored using ε_340–380(NADH)_ = 4.81 mM^−1^ cm^−1^ [23]. Catalysis was initiated by addition of NADH and maximal rates determined by linear regression. NADH:O_2_ oxidoreduction by membranes was measured similarly but using 5 μg mL^−1^ membranes in 10 mM Tris-HCl (pH 7.4) and 250 mM sucrose using 200 μM NADH and supplemented with 0.15 mM horse heart cytochrome c (Sigma-Aldrich Ltd.). Succinate:O_2_ oxidoreduction was measured using 40 μg mL^−1^ membranes in 5 mM succinate in 10 mM Tris-HCl (pH 7.4) as described previously [26]. Control experiments included NaCl (to match the ionic strength) or DMSO, as appropriate.

### Cell lines

143B (CRL-8303 from ATCC), HepG2 (85011430 from The Health Protection Agency), and MDBK (CCL-22 from ATCC) cells were grown on Dulbecco’s modified Eagle’s medium (DMEM) supplemented with 10 % fetal bovine serum (FBS, Thermo Fisher Scientific) at 37 °C in 5 % CO_2_. All cells were confirmed as negative for mycoplasma.

### Oxygen consumption rate measurements on intact and permeabilized cells

Oxygen consumption rates (OCRs) were measured using a Seahorse XF96 extracellular flux analyzer at 37 °C. For intact cell measurements, 1.4 × 10^4^ 143B, HepG2, or MDBK cells were plated (per well) in DMEM containing 10 % FBS into Seahorse Bioscience Inc. XF96 plates and incubated for ~12 h at 37 °C in 5 % CO_2_. Then, the medium was exchanged for assay buffer containing DMEM, 4.5 g L^−1^ glucose, 1 mM pyruvate, 32 mM NaCl, 2 mM GlutaMAX, 15 mg L^−1^ phenol red, and 20 mM HEPES (pH 7.4 at 37 °C) and the cells placed in a CO_2_-free incubator at 37 °C for 30 min. Basal oxygen consumption rates were established before the addition of biguanides at one-tenth of their IC_50_ (IC_50_/10), and followed for ~ 6 h before the addition of rotenone (2 μM).

For permeabilized cell experiments, cells were seeded into XF96 plates at 0.9–1.1 × 10^4^ per well and incubated for 48 h at 37 °C in 5 % CO_2_. Then, the growth medium was exchanged for assay buffer containing 3 nM plasma membrane permeabilizer ‘PMP’ (Seahorse Biosciences Inc.), 10 mM glutamate, 10 mM malate, 220 mM mannitol, 70 mM sucrose, 10 mM KH_2_PO_4_, 5 mM MgCl_2_, 1 mM EGTA, 0.2 % fatty acid-free bovine serum albumin (BSA), and 2 mM HEPES (pH 7.4 at 37 °C). The permeabilized cells were incubated with 10 mM glutamate and 10 mM malate for 25 min prior to the addition of biguanides at IC_50_/5 or IC_50_. The biguanides were allowed to accumulate for 30 min, then respiration was uncoupled by the addition of 4 mM ADP. Rotenone was added (2 μM) at the end of the experiment.

Rotenone-sensitive OCRs were calculated by subtracting the rotenone-insensitive rates (determined at the end of the experiment). OCRs were normalized by dividing by the OCR recorded immediately prior to the addition of biguanide. For rate of uptake calculations, the OCRs at time points after biguanide addition were divided by the OCRs at the same time points in control experiments. Data from wells with failed port injections were excluded from the analyses. The respiratory control ratio (RCR) values (state III versus state IV respiration) were ~11 and ~6 for permeabilized MDBK and 143B cells, respectively.

### OCR measurements on isolated mitochondria

Isolated mitochondria were monitored using a Seahorse XF96 extracellular flux analyzer at 32 °C. Mouse heart mitochondria, and mouse and rat liver mitochondria were plated into XF96 plates at 4 and 16–20 μg per well, respectively, in 220 mM mannitol, 70 mM sucrose, 10 mM KH_2_PO_4_, 5 mM MgCl_2_, 1 mM EGTA, 0.2 % fatty acid-free BSA, and 2 mM HEPES (pH 7.4 at 37 °C) with 10 mM glutamate and 10 mM malate and adhered by centrifugation (2000 × g, 20 min, 4 °C). The medium was supplemented with a further 5 mM glutamate and 5 mM malate and the mitochondria incubated for 15 min. Then two baseline readings were taken before the biguanides were added at IC_50_/5. Biguanides were allowed to accumulate for 15–20 min before respiration was uncoupled by the addition of 4 mM ADP. Rotenone (2 μM) was added at the end of the experiment. Data from wells with failed port injections were excluded from the analyses. The RCR values (state III versus state IV respiration) were 3.4, 4.8, and 4.6 for mouse heart, rat liver, and mouse liver, respectively.

### Membrane potential measurements

143B cells were seeded at 5 × 10^5^ cells per 10 cm^2^ well in DMEM containing 10 % FBS. On the following day they were treated with 34 μM phenformin (IC_50_/10) for 6.5 h then washed once with phosphate-buffered saline (PBS) and detached using TrypLE (Thermo Fisher Scientific). The detached cells from each well were incubated in PBS containing 25 pg/mL tetramethylrhodamine, methyl ester (TMRM) and 20 μg/mL Hoescht stain for 20 min at 37 °C then washed twice and re-suspended in 200 μL PBS. TMRM fluorescence was detected using a NucleoCounter® NC 3000 cytometer with excitation at 530 nm and emission at 575 nm, with the Hoescht stain used to check for consistent cell density between samples.

### Analysis of AMPK activation

MDBK cells were seeded at 1 × 10^6^ cells per 6 cm dish in DMEM containing 10 % FBS. Cells were then serum starved in DMEM containing 25 mM HEPES for 6 h before biguanides were added at IC_50_/10. After 18 h cells were harvested by a rapid lysis procedure [27]: cells were rinsed with PBS, then lysed using ice-cold buffer containing 50 mM HEPES, 1 mM EDTA, 10 % glycerol, 50 mM NaF, 5 mM sodium pyrophosphate, 1 % Triton-X100, 1 mM dithiothreitol (DTT), and a protease inhibitor cocktail (Roche). The cell lysate was centrifuged at 14,000 × g for 20 min at 4 °C and the protein concentration of the supernatant determined by bicinchoninic acid assay. The lysate was analyzed using Novex 3–12 % bis-Tris gels run in 3-(N-morpholino)propanesulfonic acid (MOPS) buffer (20 μg of protein per lane) followed by transfer to low fluorescence polyvinylidene fluoride membranes by wet transfer. Primary rabbit antibodies for AMPK-β (57C12), AMPK-α (phospho-Thr172) (40H9), and acetyl-coA carboxylase (phospho-Ser79) (Cell Signaling Technology) were incubated overnight with the membrane (1:1000 dilution) in Odyssey blocking buffer at 4 °C. A fluorescent secondary antibody was used (IRDYE800CW from LI-COR Biosciences, 1:20,000 dilution) with a LI-COR Odyssey imaging system.

### Partition coefficients

Octanol/PBS partition coefficients were measured by the shake-flask method [28] at 32 °C, pH 7.4.

### Statistical methods

Experimental values are reported as mean averages ± standard deviation (SD) of technical replicates for large sample sizes and mean averages ± standard error of the mean (SEM) of technical replicates for small sample sizes. Data were analyzed by the unpaired two-tailed Student’s *t* test. IC_50_ values were determined using the standard dose-effect relationship (activity (%) = 100 × IC_50_ / (IC_50_ + [inhibitor]^*m*^) [29] with the Hill Slope (*m*) set to unity for purified complex I) and are reported with 95 % confidence intervals.

## Results and discussion

### Experimental strategy

**Fig. 1:**
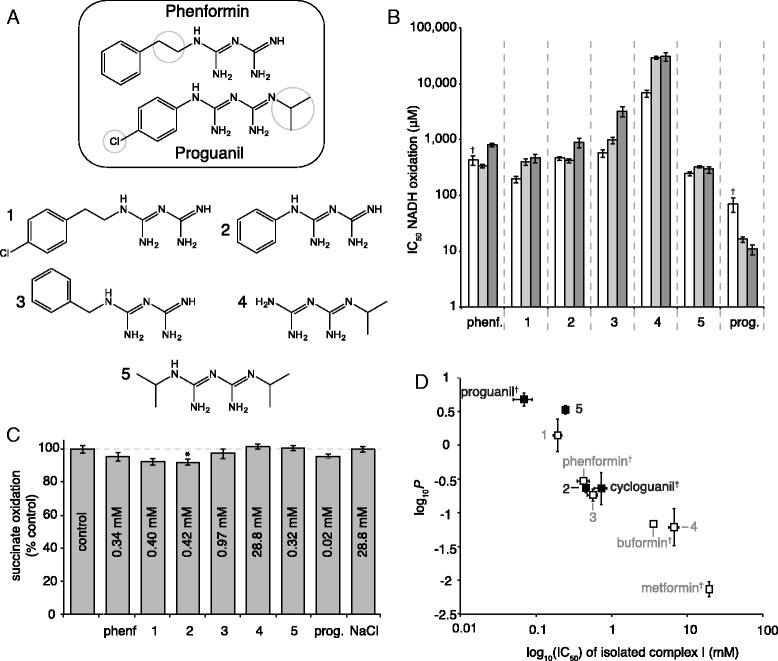
Characterization of the effects of seven biguanides on mitochondrial respiration. **a** Differences in molecular structure between phenformin and proguanil and the five test compounds (all compounds shown in the neutral form). **b** Biguanide IC_50_ values for inhibition of NADH:decylubiquinone oxidoreduction by isolated bovine heart complex I (*white*), and NADH:O_2_ oxidoreduction by bovine heart (*light gray*) and mouse heart (*dark gray*) mitochondrial membranes. The data from three or four technical replicates were fit using Prism, and presented as best-fit IC_50_ values with 95 % confidence intervals. **c** Inhibition of succinate:O_2_ oxidoreduction in bovine mitochondrial membranes by biguanides at concentrations equivalent to the IC_50_ values for NADH:O_2_ oxidoreduction. Data are mean values ± standard error of the mean (SEM) (*n* = 3); *p ≤ 0.05. **d** Correlation between the IC_50_ values for isolated bovine complex I and the octanol:PBS partition coefficients (log_10_ *P* values) of the biguanides. Compounds that inhibit cellular and mitochondrial respiration strongly (see main text) are shown with *open symbols* and *gray text*, and those that do not with *closed symbols* and *black text*. The dagger (†) indicates values reported previously [4]. Data for log_10_ *P* are mean values ± SEM (*n* = 3). Individual data points for panels **b**, **c**, and **d** are provided in Additional file 1. *phenf.* phenformin, *prog.* proguanil

To delineate the effects that determine whether biguanides are able to cross the mitochondrial inner membrane we focused on phenformin and proguanil. They have similar partition coefficients [4], so their passive transport across the membrane should be comparable; however, they are taken up into the mitochondrial matrix with very different rates and to very different extents. Both compounds are effective inhibitors of isolated complex I, but only phenformin inhibits complex I in mitochondria [4]. The molecular structure of proguanil differs from that of phenformin in four ways (Fig. 1a): its phenyl ring is chlorinated; it has no linker between the phenyl ring and the biguanide moiety (phenformin has a two-carbon linker); its biguanide moiety is bis-substituted (phenformin is mono-substituted); and it contains an isopropyl substitution. Five biguanides were designed to test each difference (Fig. 1a): 2-[2-(4-chlorophenyl)ethyl]-1-(diaminomethylidene)guanidine) (chlorophenformin, compound 1) tests the effect of adding a chlorine to the phenyl ring; phenyl biguanide (compound 2) and 2-benzyl-1-(diaminomethylidene)guanidine (benzyl biguanide, compound 3) test the effect of the linker between the phenyl ring and the biguanide moiety; 1-(diaminomethylidene)-2-(propan-2-yl)guanidine (isopropyl biguanide, compound 4) tests the effect of the isopropyl derivative; and N-(propan-2-yl)-1-3-(propan-2-yl)carbamimidamidomethanimidamide (bis-isopropyl biguanide, compound 5) tests the effect of bis-substituting the biguanide functionality.

### Biguanide effects on isolated complex I and mitochondrial membranes

All seven compounds described were effective inhibitors of NADH:decylubiquinone oxidoreduction by isolated bovine complex I, as well as of NADH:O_2_ oxidoreduction (catalysis by complexes I-III-IV) by bovine and mouse heart mitochondrial membranes (Fig. 1b and Additional file 1), and the IC_50_ values for each biological system were comparable. Subsequent assays on human cell lines were based on the IC_50_ values from bovine heart membranes, and assays on mouse mitochondria on the IC_50_ values from mouse heart membranes. Assays of succinate:O_2_ oxidoreduction (catalysis by complexes II-III-IV) by bovine membranes, using concentrations equivalent to the IC_50_ values for NADH:O_2_ oxidoreduction, confirmed the inhibition is specific to complex I (Fig. 1c and Additional file 1). Finally, partition coefficients that describe compound hydrophobicity were measured for each compound at pH 7.4 (at which pH all the biguanides are positively charged). A correlation between inhibitory efficacy and hydrophobicity was observed (Fig. 1d and Additional file 1) that extends and confirms that observed previously with a smaller number of compounds [4]. Improved transmembrane diffusion as a result of their greater hydrophobicity has previously been suggested as a reason why phenformin and buformin are more potent inhibitors of cellular respiration than metformin [30], but our data explain that this correlation is observed because the more hydrophobic compounds are better complex I inhibitors.

### Biguanide effects on oxygen consumption by cells and mitochondria

**Fig. 2:**
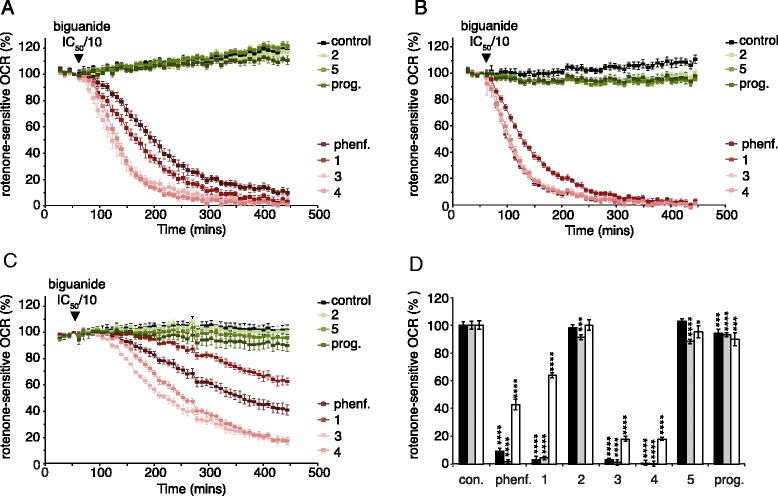
Biguanide inhibition of rotenone-sensitive respiration in intact cells. Panels **a**–**c** show the progression of rotenone-sensitive oxygen consumption rates (*OCRs*) in 143B (**a**), HepG2 (**b**), and MDBK (**c**) cells following addition of biguanides. The traces are the means ± standard deviation (SD) of multiple traces. For 143B, *n* = 9 for control and proguanil; *n* = 11 for phenformin and compounds 2 and 5; and *n* =12 for compounds 1, 3, and 4. For HEP G2, *n* = 9 for control and proguanil; *n* = 11 for phenformin and compounds 2 and 3; and *n* = 12 for compounds 1, 4, and 5. For MDBK, *n* = 8 for control; *n* = 7 for compounds 1 and 5; and *n* = 6 for phenformin, proguanil, and compounds 2, 3, and 4. Biguanide concentrations were set to IC_50_/10: 34 μM phenformin; 40 μM compound 1, 42 μM compound 2; 97 μM compound 3; 2.88 mM compound 4; 32 μM compound 5; and 2 μM proguanil. **d** Rotenone-sensitive OCRs in 143B (*black*), HepG2 (*gray*), and MDBK (*white*) cells after 6 h of biguanide incubation (% of the control, error bars show propagated SD). Individual data points for panel **d** are provided in Additional file 1. *con.* control, *phenf.* phenformin, *prog.* proguanil

All the compounds studied inhibit isolated complex I, so inhibition of oxygen consumption by the respiratory chain can be exploited as a marker for their presence and concentration in the mitochondrial matrix, the compartment containing the complex I biguanide-binding site. Notably, this approach to evaluating the matrix biguanide concentration avoids the need for radioactive biguanides, estimation of matrix volume, or lengthy isolation protocols. To account for the wide range of complex I IC_50_ values observed across the compound series, concentrations relative to the individual IC_50_ values were used. Biguanides were added to cells at one-tenth of their IC_50_ concentrations, and the rotenone-sensitive OCRs monitored for 6 h. At IC_50_/10 a biguanide that does not enter mitochondria does not inhibit respiration, whereas a biguanide that can enter the matrix accumulates based on its intrinsic positive charge and the membrane potential and so progressively inhibits respiration. Experiments with human osteosarcoma 143B (Fig. 2a) and hepatocarcinoma HepG2 cells (Fig. 2b) revealed that compounds 1, 3, and 4 inhibit respiration (like phenformin they enter mitochondria), but compounds 2 and 5 do not (like proguanil they do not enter mitochondria). The same pattern was observed in experiments using a kidney cell line from *Bos taurus* (MDBK) (Fig. 2c) although the drugs were much slower to take effect. Thus, after 6 h of exposure, 143B and HepG2 cells were inhibited to a similar level, and MDBK cells were less inhibited (Fig. 2d and Additional file 1).

**Fig. 3:**
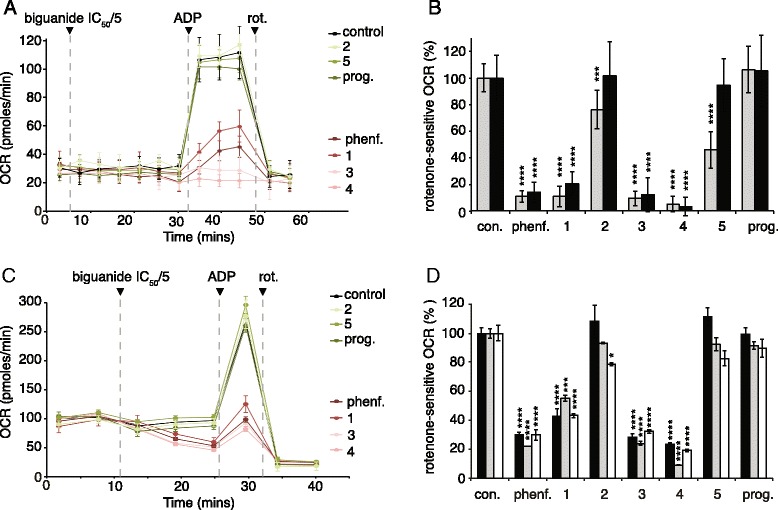
Biguanide inhibition of rotenone-sensitive respiration in permeabilized cells and isolated mitochondria. **a** MDBK permeabilized cells treated with biguanides at IC_50_/5 for 30 min before ADP was added to stimulate the rates. Data show means ± standard deviation (SD) (*n* = 16 for control, *n* = 5 for proguanil and compound 2, *n* = 6 for compound 3, *n* = 7 for compound 1, and *n* = 8 for phenformin and compounds 4 and 5). **b** ADP-stimulated rotenone-sensitive oxygen consumption rates (*OCRs*) in permeabilized cells (% of the control, error bars show SD values). For permeabilized 143B cells: *n* = 10 for control, compounds 1, 2, and 4; *n* = 11 for phenformin and compound 3; *n* = 8 for compound 5; and *n* = 9 for proguanil. *Black*, data taken from Fig. 3a; *gray*, equivalent data from permeabilized 143B cells but using biguanide concentrations equal to IC_50_. **c** Mouse heart mitochondria respiring on glutamate and malate were exposed to biguanides for 15 min before ADP was added to stimulate the rates. Biguanides concentrations were set to IC_50_/5 (using the IC_50_ values for mouse heart mitochondrial membranes): 0.16 mM phenformin, 0.094 mM compound 1, 0.17 mM compound 2, 0.63 mM compound 3, 6 mM compound 4, 0.058 mM compound 5, and 0.002 mM proguanil. Data show means ± standard error of the mean (SEM) (*n* = 8 for all compounds and *n* = 12 for control). **d** Inhibition of the ADP-stimulated rotenone-sensitive OCR in mitochondria (% of the control; mean ± SEM. For mouse liver mitochondria: *n* = 5 for control; *n* = 3 for compounds 1, 2, and 4; and *n* = 4 for all other compounds. For rat liver: *n* = 5 for control, *n* = 3 for proguanil and compound 5, and *n* = 4 for all other compounds. *Black*, data taken from Fig. 3c; *gray*, rat liver mitochondria; *white*, mouse liver mitochondria. For panels b and d, ****p ≤ 0.0001, ***p ≤ 0.001, **p ≤ 0.01, *p ≤ 0.05. Individual data points for panels b and d are provided in Additional file 1. *con.* control, *phenf.* phenformin, *prog.* proguanil, *rot.* rotenone

The data on intact cells suggest the presence of a selectivity barrier at either the plasma membrane and/or the mitochondrial inner membrane. Therefore, the compounds were tested at concentrations of IC_50_/5 on MDBK cells in which the plasma membrane had been permeabilized (Fig. 3a, b), and on isolated mitochondria from mouse heart, mouse liver, and rat liver (Fig. 3c, d). The same pattern of inhibition was observed in all cases, showing that transport across the mitochondrial inner membrane is selective. Even much higher (IC_50_) concentrations of compounds 2, 5, and proguanil elicited only limited inhibition of the OCR in permeabilized 143B cells (Fig. 3b and Additional file 1). Furthermore, much less inhibition was observed in an equivalent experiment using mouse heart mitochondria in which ADP was added at the same time as phenformin [oxygen consumption was 85 ± 10 % of the control value (*n* = 8 with phenformin and *n* = 6 for control, see Additional file 2), compared to 30 % in the absence of ADP (see Fig. 3d and Additional file 1)], confirming that biguanide accumulation into mitochondria depends on the membrane potential [12]. Finally, the inhibition of the OCR observed in intact mitochondria respiring on glutamate and malate confirms that it is due to inhibition of complex I, not mitochondrial glycerol-3-phosphate dehydrogenase (mGPD) [31]. mGPD has also been proposed as a target for biguanides [31] and could contribute to the inhibition of the OCR in intact cells because it couples oxidation of the NADH produced by glycolysis in the cytoplasm to reduction of O_2_ by complex IV. However, the malate/aspartate shuttle also performs this function, and mGPD does not contribute to oxygen consumption by isolated mitochondria because they lack any connection with cytoplasmic processes.

### The molecular determinants of biguanide uptake

Our data clearly delineate compounds 1, 3, and 4 that can cross both the plasma and mitochondrial inner membrane (like phenformin) from compounds 2 and 5 that cannot (like proguanil). Thus, conjugation of a phenyl ring directly to the biguanide moiety (compound 2) and bis-substitution of the biguanide (compound 5) prevent biguanides from accessing the matrix and inhibiting respiration; both features are present in proguanil. A chloro-group on the ring (compound 1) and the iso-propyl group itself (compound 4), which are also present in proguanil, do not prevent inhibition. The inhibition observed previously from alkyl biguanides, including the anti-diabetic compounds metformin and buformin [4, 32], is fully consistent with our results.

### Rate and extent of biguanide uptake

Biguanide uptake into mitochondria depends on the mitochondrial membrane potential. However, once inside mitochondria, biguanides inhibit complex I and thus may decrease the membrane potential, resulting in a negative feedback loop that will eventually balance the two effects. To evaluate the effects of phenformin on membrane potential, 143B cells were treated with phenformin at IC_50_/10 to match the experiment in Fig. 2a, and the membrane potential was evaluated using the fluorescence of TMRM. After 6.5 h, the TMRM fluorescence was 22.4 ± 2.9 % (*n* = 3) of the control value, compared to 13.43 ± 1.7 % (*n* = 3) in cells treated with 1 μM of the uncoupler carbonyl cyanide-4-(trifluoromethoxy)phenylhydrazone (FCCP) (Additional file 3). Although the TMRM fluorescence is not a quantitative measure, it is clear that phenformin treatment induces a decrease in the membrane potential. We previously found that, in addition to their effects on complex I, biguanides also directly inhibit ATP hydrolysis by F_1_F_o_ ATP synthase, hindering the use of ATP hydrolysis to support the membrane potential [4] when the respiratory chain is inhibited.

**Fig. 4:**
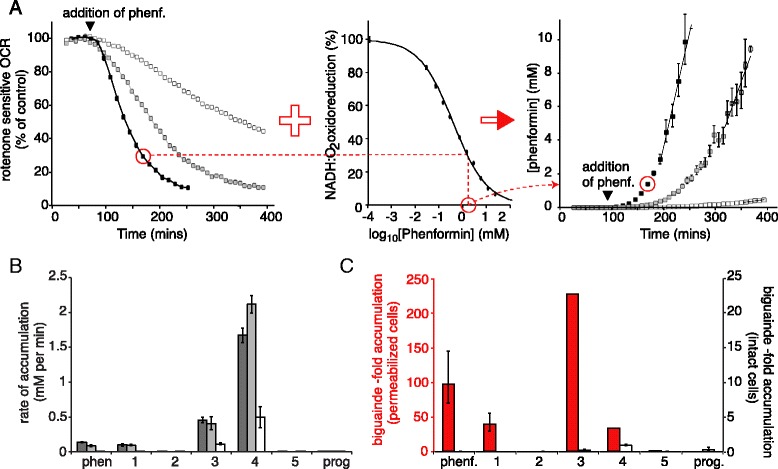
Determination of matrix phenformin concentrations in cells. **a** Oxygen consumption rate (*OCR*) as a percentage of the control for HepG2 (*black*), 143B (*gray*), and MDBK (*white*) cells, taken from Fig. 2 (*left*, 34 μM phenformin = IC_50_/10), is combined with the IC_50_ curve for phenformin inhibition of complex I in bovine mitochondrial membranes (*middle*), providing estimates for the matrix phenformin concentration (*right*). **b** Maximal rates of biguanide uptake into the matrix were calculated by linear regression from the data in Fig. 2a–c. *Dark gray*, 143B; *light gray*, HepG2; *white*, MDBK. **c** Biguanide concentration in the matrix relative to in the extracellular medium after 30 min in intact (*white*) MDBK and permeabilized (*red*) MDBK cells. For intact cells: *n* = 8 for control; *n* = 7 for compounds 1 and 5; and *n* = 6 for phenformin, proguanil, and compounds 2, 3, and 4. For permeabilized cells: *n* = 16 for control; *n* = 8 for phenformin and compounds 4 and 5; *n* = 7 for compound 1; *n* = 6 for compound 3; and *n* = 5 for proguanil and compound 2. The values for compounds 3 and 4 are underestimates as inhibition exceeded 90 %. Data show means ± standard error of the mean. Individual data points for panel c are provided in Additional file 1. *phenf.* phenformin, *prog.* proguanil

In order to evaluate how the matrix biguanide concentration varies in intact cells, inhibition of the OCR was exploited as a real-time marker by comparison with the IC_50_ data from complex I in membranes (Fig. 4a). Note that we are unable to include the effects of the decreased membrane potential (which acts to increase the OCR, opposing the inhibition) in our calculation, so the calculated values are underestimates of the true matrix concentration. Figure 4a shows how the estimated matrix phenformin concentration evolves over time, with maximal rates of accumulation of 136, 85, and 3.2 μM min^−1^ for HepG2, 143B, and MDBK cells, respectively. Figure 4b compares rates of uptake of each compound into each cell line, confirming that the weak inhibition in MDBK cells (Fig. 2c) is due to slow biguanide uptake. For the four biguanides (phenformin and compounds 1, 3, and 4) that are taken up into mitochondria, the estimated rates range from >1.5 mM min^−1^ for compound 4 into 143B and HepG2 cells (3 mM in the extracellular medium) to 1 μM min^−1^ for compound 1 into MDBK cells (37 μM in the extracellular medium); because the biguanides are present in the medium at different absolute concentrations, the rates are not directly comparable. Figure 4c compares the extent of accumulation after 30 min of biguanide exposure for intact and permeabilized MDBK cells, calculated in the same way (see also Additional file 1). On this timescale the intramitochondrial concentrations in intact cells hardly reach the concentration in the extracellular medium, whereas in permeabilized cells the compounds accumulate significantly into mitochondria, with compound 3 accumulating more than 200-fold. Therefore, the slow uptake of the mitochondria-permeant biguanides observed in MDBK cells is due to slow transport across the plasma membrane.

### Is biguanide transport protein mediated or determined by membrane solubility?

The transport of metformin across the plasma membrane by OCTs is widely accepted, but more hydrophobic biguanides like phenformin have been widely considered to cross biological membranes by passive transport [6, 19, 20]. The most hydrophobic biguanide tested here, proguanil, is known to cross the plasma membrane into liver cells because it is converted to cycloguanil by P450s [33], but it does not reach the mitochondrial matrix to inhibit complex I. Here, we have shown that selective transport across the inner membrane both prevents proguanil (and compounds 2 and 5) from accumulating in mitochondria, and facilitates the rapid uptake of phenformin (and compounds 1, 3, and 4). This selectivity strongly suggests that transport is protein mediated. By extension, the different uptake rates between cell lines can be ascribed to different expression levels of transporter proteins, particularly in the plasma membrane. Here, we observed the slowest uptake in bovine kidney cells, and it has been observed that biguanide uptake into human HEK293 kidney cells is very slow unless OCTs are overexpressed [34]. Hepatic cells (like HepG2) are known to have high OCT1 expression levels for biguanide uptake and, conversely, kidney cells have high MATE1 expression levels for biguanide efflux [16]. Although the simplest explanation for our data is selective biguanide uptake into mitochondria, the influence of highly active and selective efflux processes cannot be excluded.

### Activation of AMPK by biguanides

**Fig. 5:**
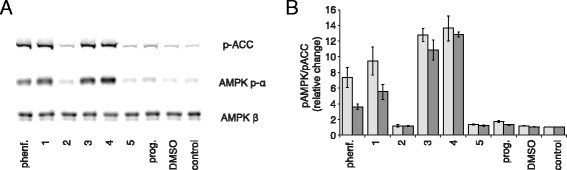
AMP-activated protein kinase (*AMPK*) activation by biguanides in MDBK cells. **a** Western blot against phospho-acetyl-coA carboxylase (*p-ACC*), phospho-AMPK-α (*AMPK p-α*), and AMPK-β. Cells were incubated for 18 h with biguanide concentrations equivalent to IC_50_/10. **b** Quantification of western blot band intensities; the signals from p-ACC and AMPK p-α were normalized to the signals for AMPK-β, and then further normalized to the control values. Data show the mean of independent experiments ± standard error of the mean (*n* = 3). Individual data points for panel B are provided in Additional file 1. *phenf.* phenformin, *prog.* proguanil

The anti-hyperglycemic and anti-proliferative activities of biguanides have been linked to the activation of AMPK [7, 8] and so the ability of each biguanide to activate AMPK was investigated in MDBK cells. Figure 5 shows the levels of activation of AMPK (and its downstream effector acetyl-coA carboxylase, ACC) achieved following 18-h treatments with IC_50_/10 biguanide concentrations. The antibodies to AMPK-α and ACC detect the phosphorylated forms (with the detection of AMPK-β as a control) and AMPK activation was observed only for those biguanides that inhibit mitochondrial respiration. Indeed, the slightly lower activation observed for phenformin and compound 1 is consistent with their less efficient inhibition in this cell line (Fig. 2c).

Our data indicate that access to the mitochondrial matrix is necessary for biguanide-mediated AMPK activation, so it is unlikely that an extra-mitochondrial enzyme is the primary target responsible for AMPK-related downstream effects of biguanide treatments. AMP deaminase in the cytoplasm and mGPD in the intermembrane space have both been proposed as primary extra-mitochondrial targets of metformin [31, 35]; our results suggest that the inhibition of neither of them is linked to the activation of AMPK. Consistent with this picture, acute metformin treatments were observed to inhibit mGPD and cause plasma glucose to drop, but not to activate AMPK [31], whereas long-term metformin treatments did activate AMPK. Whether the anti-hyperglycemic effects of metformin (and phenformin) are mediated by AMPK activation [7, 36, 37] or by alternative signaling pathways [2, 38] (or both) is thus still debated. The anti-proliferative effects of biguanides have been more closely linked to inhibition of complex I, as they were ablated by overexpression of the yeast alternative NADH:ubiquinone oxidoreductase NDI1 [12]; they may be mediated by mTORC1 in either an AMPK-dependent or independent manner [3, 11].

## Conclusions

All the biguanides tested inhibit mitochondrial complex I, but only some biguanides inhibit mitochondrial respiration in vivo. Here, we have identified the specific molecular features that control the uptake of substituted biguanides into mitochondria and allow them to target complex I: direct conjugation of a phenyl group and bis-substitution of the biguanide moiety prevent uptake into mitochondria, irrespective of the compound hydrophobicity. The high selectivity suggests that biguanide uptake into mitochondria is protein mediated, and is not by passive diffusion. Only biguanides that enter mitochondria and inhibit complex I activate AMP kinase. Our results can be applied in the design of new complex I-targeted biguanides with improved anti-proliferative activity [39], and to assist in deconvoluting the targets and mechanisms of action of anti-hyperglycemic biguanides like metformin.

## Abbreviations

ACC, acetyl-coA carboxylase; AMPK, AMP-activated protein kinase; BSA, bovine serum albumin; DMEM, Dulbecco’s modified Eagle’s medium; FBS, fetal bovine serum; FCCP, carbonyl cyanide-4-(trifluoromethoxy)phenylhydrazone; MATE, multidrug and toxin extrusion proteins; mGPD, mitochondrial glycerol-3-phosphate dehydrogenase; OCR, oxygen consumption rate; OCT, organic cation transporter; PBS, phosphate-buffered saline; RCR, respiratory control ratio; SD, standard deviation; SEM, standard error of the mean; SERT, serotonin transporter; TMRM, tetramethylrhodamine, methyl ester

## Additional files

Additional file 1: Supporting information and individual data points for figures 1b, 1c, 1d, 2d, 3b, 3d, 4c, and 5b. (XLSX 152 kb) Additional file 2: Individual data points for mouse heart mitochondria simultaneously uncoupled with ADP and inhibited with phenformin. (XLSX 56 kb) Additional file 3: Data for TMRM measurements on 143B cells. (XLSX 47 kb)

## References

[1] Luengo A, Sullivan LB, Vander Heiden MG (2014). Understanding the complex-I-ty of metformin action: limiting mitochondrial respiration to improve cancer therapy. BMC Biol..

[2] Foretz M, Guigas B, Bertrand L, Pollak M, Viollet B (2014). Metformin: from mechanisms of action to therapies. Cell Metab..

[3] Bost F, Decoux-Poullot A-G, Tanti JF, Clavel S (2016). Energy disruptors: rising stars in anticancer therapy?. Oncogenesis..

[4] Bridges HR, Jones AJY, Pollak MN, Hirst J (2014). Effects of metformin and other biguanides on oxidative phosphorylation in mitochondria. Biochem J..

[5] Owen MR, Halestrap AP (1993). The mechanisms by which mild respiratory chain inhibitors inhibit hepatic gluconeogenesis. Biochim Biophys Acta..

[6] Owen MR, Doran E, Halestrap AP (2000). Evidence that metformin exerts its anti-diabetic effects through inhibition of complex 1 of the mitochondrial respiratory chain. Biochem J..

[7] Shaw RJ, Lamia KA, Vasquez D, Koo S-H, Bardeesy N, Depinho RA (2005). The kinase LKB1 mediates glucose homeostasis in liver and therapeutic effects of metformin. Science..

[8] Hawley SA, Ross FA, Chevtzoff C, Green KA, Evans A, Fogarty S (2010). Use of cells expressing γ-subunit variants to identify diverse mechanisms of AMPK activation. Cell Metab..

[9] Ben Sahra I, Laurent K, Loubat A, Giorgetti-Peraldi S, Colosetti P, Auberger P (2008). The antidiabetic drug metformin exerts an antitumoral effect in vitro and in vivo through a decrease of cyclin D1 level. Oncogene..

[10] Zakikhani M, Dowling R, Fantus IG, Sonenberg N, Pollak M (2006). Metformin is an AMP kinase-dependent growth inhibitor for breast cancer cells. Cancer Res..

[11] Kalender A, Selvaraj A, Kim SY, Gulati P, Brûlé S, Viollet B (2010). Metformin, independent of AMPK, inhibits mTORC1 in a rag GTPase-dependent manner. Cell Metab..

[12] Wheaton WW, Weinberg SE, Hamanaka RB, Soberanes S, Sullivan LB, Anso E (2014). Metformin inhibits mitochondrial complex I of cancer cells to reduce tumorigenesis. eLife..

[13] He L, Wondisford FE (2015). Metformin action: concentrations matter. Cell Metab..

[14] Liang X, Chien H-C, Yee SW, Giacomini MM, Chen EC, Piao M (2015). Metformin is a substrate and inhibitor of the human thiamine transporter, THTR-2 (SLC19A3). Mol Pharm..

[15] Han TK, Proctor WR, Costales CL, Cai H, Everett RS, Thakker DR (2015). Four cation-selective transporters contribute to apical uptake and accumulation of metformin in Caco-2 cell monolayers. J Pharmacol Exp Ther..

[16] Lickteig AJ, Cheng X, Augustine LM, Klaassen CD, Cherrington NJ (2008). Tissue distribution, ontogeny and induction of the transporters Multidrug and toxin extrusion (MATE) 1 and MATE2 mRNA expression levels in mice. Life Sci..

[17] Alnouti Y, Petrick JS, Klaassen CD (2006). Tissue distribution and ontogeny of organic cation transporters in mice. Drug Metab Dispos..

[18] Graham GG, Punt J, Arora M, Day RO, Doogue MP, Duong JK (2011). Clinical pharmacokinetics of metformin. Clin Pharmacokinet..

[19] Shackelford DB, Abt E, Gerken L, Vasquez DS, Seki A, Leblanc M (2013). LKB1 inactivation dictates therapeutic response of non-small cell lung cancer to the metabolism drug phenformin. Cancer Cell..

[20] Pollak M (2013). Potential applications for biguanides in oncology. J Clin Invest..

[21] Lamhonwah A-M, Tein I (2006). Novel localization of OCTN1, an organic cation/carnitine transporter, to mammalian mitochondria. Biochem Biophys Res Commun..

[22] Shitara Y, Nakamichi N, Norioka M, Shima H, Kato Y, Horie T (2013). Role of organic cation/carnitine transporter 1 in uptake of phenformin and inhibitory effect on complex I respiration in mitochondria. Toxicol Sci..

[23] Sharpley MS, Shannon RJ, Draghi F, Hirst J (2006). Interactions between phospholipids and NADH:ubiquinone oxidoreductase (complex I) from bovine mitochondria. Biochemistry..

[24] Chappell JB, Hansford RG. Subcellular components: preparation and fractionation. 2nd ed. Birnie GD, ed. London: Butterworth; p. 1972:77–91.

[25] Fernández-Vizarra E, Ferrín G, Pérez-Martos A, Fernández-Silva P, Zeviani M, Enríquez JA (2010). Isolation of mitochondria for biogenetical studies: an update. Mitochondrion..

[26] Jones AJY, Hirst J. A spectrophotometric coupled enzyme assay to measure the activity of succinate dehydrogenase. Anal Biochem. 2013;442:19–23. 10.1016/j.ab.2013.07.018 PMC3783901 23886887

[27] Daniel T, Carling D. Functional analysis of mutations in the γ2 subunit of AMP-activated protein kinase associated with cardiac hypertrophy and Wolff-Parkinson-White syndrome. J Biol Chem. 2002;277:51017–24. 10.1074/jbc.M207093200 12397075

[28] Danielsson L-G, Zhang Y-H (1996). Methods for determining *n*-octanol-water partition constants. Trends Anal Chem..

[29] Holford NH, Sheiner LB (1981). Understanding the dose-effect relationship. Clin Pharmacokinet..

[30] Dykens JA, Jamieson J, Marroquin L, Nadanaciva S, Billis PA, Will Y (2008). Biguanide-induced mitochondrial dysfunction yields increased lactate production and cytotoxicity of aerobically-poised HepG2 cells and human hepatocytes in vitro. Toxicol Appl Pharmacol..

[31] Madiraju AK, Erion DM, Rahimi Y, Zhang X-M, Braddock DT, Albright RA (2014). Metformin suppresses gluconeogenesis by inhibiting mitochondrial glycerophosphate dehydrogenase. Nature..

[32] Schäfer G (1969). Site-specific uncoupling and inhibition of oxidative phosphorylation by biguanides. II. Biochim Biophys Acta..

[33] Kerb R, Fux R, Mörike K, Kremsner PG, Gil JP, Gleiter CH (2009). Pharmacogenetics of antimalarial drugs: effect on metabolism and transport. Lancet Infect Dis..

[34] Chien H-C, Zur AA, Maurer TS, Yee SW, Tolsma J, Jasper P (2016). Rapid method to determine intracellular drug concentrations in cellular uptake assays: application to metformin in organic cation transporter 1-transfected human embryonic kidney 293 cells. Drug Metab Dispos..

[35] Ouyang J, Parakhia RA, Ochs RS (2011). Metformin activates AMP kinase through inhibition of AMP deaminase. J Biol Chem..

[36] Hardie DG, Alessi DR (2013). LKB1 and AMPK and the cancer-metabolism link - ten years after. BMC Biol..

[37] Fullerton MD, Galic S, Marcinko K, Sikkema S, Pulinilkunnil T, Chen Z-P (2013). Single phosphorylation sites in Acc1 and Acc2 regulate lipid homeostasis and the insulin-sensitizing effects of metformin. Nat Med..

[38] Miller RA, Chu Q, Xie J, Foretz M, Viollet B, Birnbaum MJ (2013). Biguanides suppress hepatic glucagon signalling by decreasing production of cyclic AMP. Nature..

[39] Narise K, Okuda K, Enomoto Y, Hirayama T, Nagasawa H (2014). Optimization of biguanide derivatives as selective antitumor agents blocking adaptive stress responses in the tumor microenvironment. Drug Des Devel Ther..

